# Plasmonic Nanoceria: A Plasmon-Enhanced Nanohybrid for Rapid and Sensitive Detection of Ebola Glycoprotein

**DOI:** 10.1021/acsanm.5c01649

**Published:** 2025-04-30

**Authors:** Carissa Sutton, Kristos Baffour, Cassidy Soard, Sneha Ramanujam, Rishi Patel, Santimukul Santra, Tuhina Banerjee

**Affiliations:** † Department of Chemistry and Biochemistry, Missouri State University, 901 S. National Avenue, Springfield, Missouri 65897, United States; ‡ Department of Chemistry, College and Arts and Sciences, Pittsburg State University, 1701 S. Broadway Street, Pittsburg, Kansas 66762, United States; § Jordan Valley Innovation Center, Missouri State University, 542 N. Boonville Avenue, Springfield, Missouri 65806, United States

**Keywords:** plasmonic nanoceria, Ebola, surface plasmon resonance, viral proteins, colorimetry

## Abstract

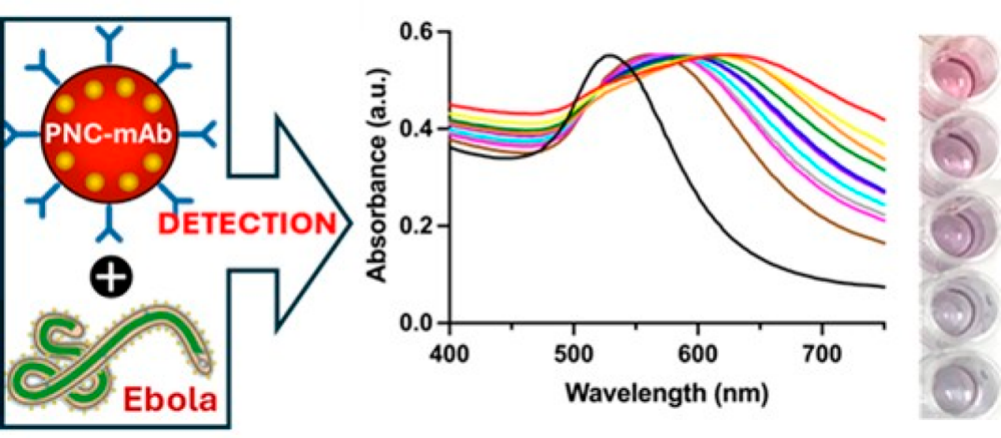

Frequent Ebola outbreaks on an unprecedented scale in resource-limited countries have resulted in higher fatality rates for the human population. Thereby, the development of a biosensor platform that can be used for point-of-care (PoC) tests and simultaneously features high sensitivity and selectivity is urgently needed. Herein, an approach for formulating multifunctional nanocomposite materials, plasmonic nanoceria (PNC), is presented, and its application as a sensing platform for the detection of Ebolavirus glycoprotein (EGP) of the Zaire strain is demonstrated. The synthetic strategy for PNC allows optical tunability, a unique approach to amplify detection sensitivity introduced by encapsulating gold nanoparticles (GNPs) within the polymeric coatings of cerium oxide nanoparticles (NC). Through altered optical characteristics of GNPs within the PNC, which include changes in localized surface plasmon resonance (SPR), higher detection sensitivity is achieved. Following surface conjugation of PNC with EGP-specific antibodies, a quantitative detection limit as low as 10 pM (0.7 ng/mL) is achieved. Moreover, antibody-functionalized PNC exhibits faster, reproducible, and highly sensitive colorimetric readouts, with a detectable SPR shift in the presence of EGP. Importantly, the limit of detection of EGP evaluated in complex sample matrices was comparable to as attained in a simple buffer. Specificity studies suggest that the developed PNC nanoplatform allows for both detection and differentiation between Ebola virus subtypes. Overall, the formulated PNC holds great potential for the rapid, ultrasensitive, and on-site detection of biomarker EGP of the Zaire strain and can be customized for the detection of other pathogens.

## Introduction

Filoviridae are a family of viruses that cause severe hemorrhagic fever among humans and other species.^1^ Two viruses belonging to this family are Ebolavirus and Marburgvirus. Among these, Ebola is known to be one of the most deadly viruses.^2^ Not all Ebola virus species infect humans; the four known ones are Zaire ebolavirus, Sudan ebolavirus, Taï Forest ebolavirus, and Bundibugyo ebolavirus.^3^ Ebola virus disease (EVD) was first discovered in 1976 with two outbreaks, one in South Sudan and the other in the Democratic Republic of Congo.^4,5^ Since then, there have been periodic outbreaks, with the most deadly one recorded in the year 2014 in West Africa. In the 2014 outbreak, 29,000 people were infected with approximately 11,000 fatalities, and 60% of the cases did not have an accurate diagnosis.^6−9^ The main reason for EVD’s high mortality rates, especially in resource-limited settings, is due to the absence of rapid and sensitive diagnostic assays.^6,10^ Additionally, EVD is highly contagious and is transmitted to people either from wild animals or through human-to-human contact.^6,7^ However, the spread of infection and high fatality rates decline tremendously if the Ebola virus (EBOV) is detected quickly and the infected patient is secluded within a day of sickness.^11−13^ Hence, fast diagnostic examinations with clinical intervention are critical for EVD management.^11^

Many diagnostic approaches with varying specificity and applicability have been proposed for the detection of EBOV.^10^ Current state-of-the-art diagnostic assays, including enzyme-linked immunosorbent assay (ELISA) and reverse transcription–polymerase chain reaction (RT-PCR), are considered to be sensitive and specific but give results within few hours, which often delays early therapeutic interventions.^14−22^ Moreover, the need for labor-intensive procedures, high-quality sample preparation, and susceptibility to contamination during amplification limits their application for on-site detection. As such, isothermal amplification methods like LAMP and RT-LAMP have been utilized to achieve a limit of detection of 70–300 copies per reaction in detecting various Ebola strains. The assays are robust, sensitive, rapid, and can detect Ebola virus RNA in the presence of minimally diluted bodily fluids within 5 to 7 min.^23^ However, they are incapable of detecting the Ebola virus at an early stage of virulence when the viral load is very low. Their efficiency is also easily interfered with by the presence of impurities.^23^

Other EBOV detection assays that have shown promise are (i) opto-fluidic biosensors based on plasmonic nanoholes, (ii) interferometric measurement techniques, (iii) luminescence resonance energy transfer strategies, and (iv) electroluminescence methods.^24−32^ Each of these platforms has its own advantages and limitations. A ferromagnetic nanoparticle (MNP)-based nanozyme strip has been proposed for the detection of EBOV. This strip detected the Ebola virus at a concentration of 1 ng/mL, 100 times more sensitive than current conventional methods.^24^ Tsang et al. achieved a detection limit at the picomolar level with a nanoporous system that comprised of BaGdF5:Yb/Er up-conversion nanoparticles (UCNPs) conjugated with the target Ebola virus oligonucleotide.^26^ Despite the specificity achieved by the nanoplatform, the prohibitive cost and the extended duration of time associated with the extraction of the oligonucleotide by a commercial kit reduced its potential for PoC settings. A novel Field-Effect Transistor (FET) biosensor was developed for rapid detection of EBOV. It detected EBOV at as low as 1 ng/mL with high selectivity.^28^ However, the sensor’s performance in plasma samples was not well-defined. In addition, multifunctional nanospheres containing gold nanoparticles and several quantum dots have been used to successfully achieve dual-signal detection of 2 ng/mL glycoprotein within 20 min.^31^ However, the merits of this report on its specificity for the Ebola virus have not been studied on relevant species, such as the Marburg virus, to distinguish it from Ebola infections. Many cost-efficient point-of-care techniques have also been established for the diagnosis of EBOV^32−34^; however, they are limited due to their low sensitivity. Hence, a platform that can offer rapid tests without compromising sensitivity is highly desirable for resource-limited settings.^35^

In this study, we present a new and straightforward method for creating a dual-readout hybrid nanostructure (PNC) with tunable plasmonic properties that allow the ultrasensitive and rapid detection of Ebolavirus glycoprotein (EGP, ∼69.3 kDa) of the Zaire strain in simple and biologically relevant matrices with little to no sample preparation. Zaire Ebola glycoprotein-specific antibodies (anti-EBOV-mAb) are conjugated onto the surface of the PNC. Each PNC contains several SPR-active gold nanoparticles that allow for the quantitative detection of the target protein at low picomolar concentrations (10 pM, ∼0.7 ng/mL) within 30 min. Results from the present findings have demonstrated the excellent analytical performance of PNC for detecting EGP of Zaire strains, even in complex biological matrices. Our approach also allows one-step detection and differentiation between different subtypes of the Ebola virus. The utility of the PNC is also demonstrated by screening candidate receptors, which the virus uses to gain entry inside the host cell. This technology could be customized for the detection of several other pathogens.

## Experimental Section

### Reagents

Hydrogen tetrachloroaurate (III) trihydrate (HAuCl_4_·3H_2_O), cerium nitrate hexahydrate (Ce(NO_3_)_3_·6H_2_O), ammonium hydroxide (NH_4_OH), phosphate buffer saline (PBS), and sodium citrate were acquired from Fisher Scientific and used as received. Polyacrylic acid (PAA) was purchased from Sigma-Aldrich. The dialysis bag (MWCO 6–8 K) was purchased from Spectrum Laboratories. Zaire Ebola glycoprotein, Sudan-Ebola glycoprotein, as well as antibody, Zika envelop protein, dengue envelop protein, TIM-1, and AXL were obtained from Alpha diagnostic. Hemagglutinin (HA) protein was obtained from Sino Biological Inc.

### Instrumentations

Hydrodynamic diameter and zeta potential of nanoparticles were measured using Malvern’s Zetasizer-ZS90. A SpectraMax M5 plate reader was used for the measurement of surface plasmon resonance. Transmission electron microscopy (TEM) images were acquired on a JEOL JEM-2100 Scanning Transmission Electron Microscope (STEM) with a Bruker Quantax 200 energy-dispersive X-ray microanalysis (EDS) system to determine the morphology and elemental composition of the synthesized nanomaterials. XPS experiments were performed on a Nexsa XPS surface analysis system from Thermo Fisher Scientific, fitted with a microfocused, monochromated, low-power, Al Kα (1486.6 eV) X-ray radiation source.

### Preparation of Cerium Oxide Nanoparticles (Nanoceria)

Nanoceria was synthesized by preparing two solutions. Solution 1 contained 0.9 g of cerium nitrate dissolved in 2.5 mL of DI water. Solution 2 contained 0.9 g of PAA in 10 mL of DI water. In a 150 mL Erlenmeyer flask, 30 mL of ammonium hydroxide was stirred at 850 rpm. The cerium salt solution was added to the ammonium hydroxide and allowed to react before the addition of the PAA solution. After continuous stirring for 24 h, a change from opaque brown to light yellow was observed. The solution was then centrifuged at a speed of 3000 rpm for 20 min, and the supernatant (NC) was separated after centrifugation and purified by dialysis for 12 h using a dialysis bag.

### Preparation of Gold Nanoparticles (GNPs)

In an Erlenmeyer flask, 2.0 mL (5.0 mM) of freshly prepared hydrogen tetrachloroaurate (III) trihydrate solution (HAuCl_4_·3H_2_O) was diluted to 17 mL with DI water and was allowed to boil with constant stirring. Once the solution started to boil, preheated 1.0% sodium citrate (1.0 mL) was added, and the reaction continued for 15 min. The successful reduction of hydrogen tetrachloroaurate (III) trihydrate solution, initiated by sodium citrate, resulted in its characteristic wine-red color solution, indicating the formation of gold nanoparticles with a corresponding wavelength maximum (λ_max_ = 520 nm). The appearance of the wine-red color was used as the end point for the reaction.

### Preparation of Plasmonic Nanoceria (PNC)

A new water-based synthetic protocol was developed for PNC synthesis, which included two steps: nanoceria synthesis and in situ GNP preparation using the Turkevich method. Briefly, a solution containing nanoceria (5.0 mM) and HAuCl4 solution (5.0 mM) was boiled for 10 min. Next, a sodium citrate solution (1.0%) was added to reduce gold(III) to gold (0) and entrapped within the PAA coatings of nanoceria. The appearance of a wine-red color from yellow marked the completion of the PNC synthesis. The resulting PNC solution was purified by dialysis and stored at 4 °C for future experiments. For TEM and EDS experiments, dilute solutions of PNC were used. These solutions were added onto commercially available copper grids (Electron Microscopy Sciences) and dried under vacuum.

### Conjugation of Anti-Zaire IgG/EGP on PNC

The concentration of the antibodies for the synthesis of the PNC conjugate and the pH for conjugation were optimized for passive immobilization of antibodies onto the PNC. In brief, PNC (OD = 1.0) solutions were adjusted to pH 9.0 with 0.1 M potassium carbonate. Subsequently, 0.1 mL of antibody (6.0 μg/mL) was added. Following mixing, the conjugate solution was incubated for 10 min at room temperature. Finally, 5% BSA was added to the conjugate mixture to block nonspecific binding and centrifuged at 8000*g* for 10 min at 4 °C to remove excess antibody and finally suspended in carbonate buffer. Confirmation of successful conjugation was done by measuring changes in size, zeta potential, and SPR.

### Procedure for PNC Detection Assay

Different solutions with increasing concentrations from 10 × 10^–12^ (M) to 1000 × 10^–12^ (M) of EGP protein were dissolved in 1X PBS and added in a 96-well plate. To these above solutions, 200 μL of the PNC conjugate (2.0 mM) was added. A negative control sample without EGP was also included. Following 30 min of incubation, UV–vis scans were collected via the SpectraMax M5 plate reader, and color transitions occurring in each sample well were imaged.

### Procedure for PNC Competition Assay

In a 96-well plate, free anti-Zaire IgG (6.0 μM) was added to different solutions of Ebola protein (EGP). Then, 200 μL of PNC-mAb (2.0 mM) was added to each well plate containing the above mixture, and UV–vis scans were collected following 30 min of incubation. A control solution containing only PNC-mAb with no spiked EGP was also included.

### Specificity and One-Step Subtype Detection Studies

A cross-reactivity study was done to detect the specificity of the PNC conjugate to EGP. Solutions of DENV, ZENV, and HA (400 pM), known protein biomarkers for dengue, Zika, and influenza infections, were included for the analysis along with EGP. A sample containing these biomarker proteins was also evaluated. To each well, 200 μL of anti-Zaire IgG PNC (PNC-mAb, 2.0 mM) was added. A control solution containing only anti-Zaire IgG PNC was included. In addition, the test sample containing 400 pM Sudan EGP was also measured. Following 30 min of incubation, SPR data and color changes occurring in each sample were analyzed.

### Receptor Screening Studies

The EGP affinity toward various receptor proteins (TIM-1, ZENV-Ab, AXL, and HSP70) was assessed. Each well containing various receptor proteins (50 μM) was incubated with 200 μL of PNC-EGP (2.0 mM).

### Procedure of PNC Detection Assay in Complex Biological Samples

To evaluate the detection sensitivity of the functional PNC in real-world samples, complex sample matrices including whole blood, urine, and serum were diluted to the desired percentages using 1X PBS (pH 7.4). Dilutions of the complex samples were performed to reduce matrix interferences. Following dilution, the samples were spiked with different amounts of EGP to get the required concentrations, and the detection assay was conducted following the same procedure described above.

## Results and Discussion

### Ebola Detection Mechanism Using Functional PNC

**Scheme 1:**
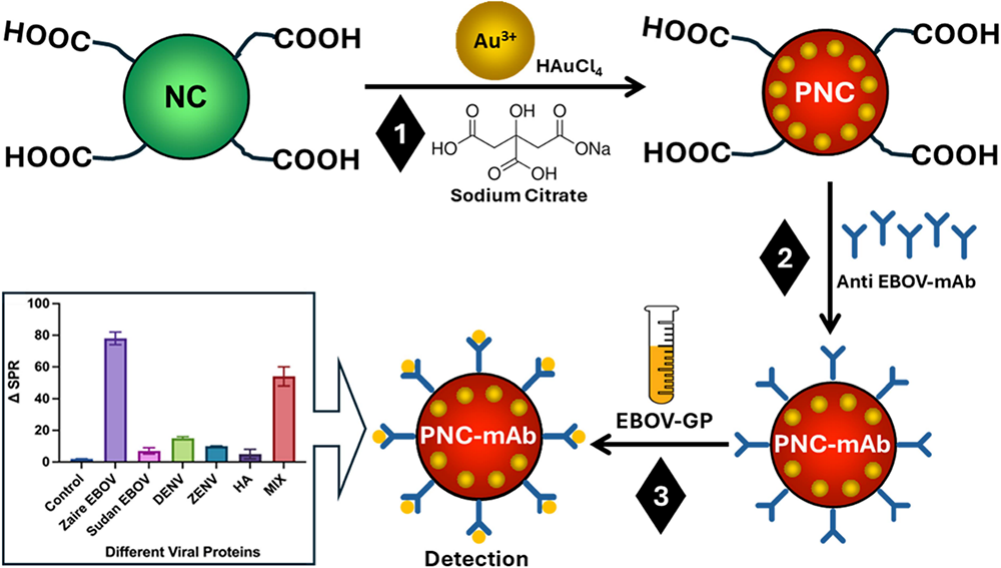
Schematic Representation of the Synthesis of Plasmonic Nanoceria (PNC) and Its Conjugate for the Specific Detection of Ebola Glycoprotein (EGP)

For maximizing the sensitivity of conventional gold nanoparticle-based colorimetric detection, in this study, a new hybrid nanomaterial, plasmonic nanoceria (PNC), was developed for the detection of Ebolavirus glycoprotein (EGP) of the Zaire strain. PNC formulation (Scheme 1) involves two distinct steps: the initial step includes the synthesis of poly(acrylic acid) (PAA)-coated cerium oxide nanoparticles referred to as nanoceria (NC),^36,37^ and the second step involves the in situ formation of GNPs inside the PAA coating of NC using the Turkevich method.^38−41^ During the synthesis of NC, the thickness of the polymer coating of cerium oxide nanoparticles is carefully optimized to achieve plasmonic tunability of the loaded GNPs. The in situ formulation of PNC and one-step encapsulation of several GNPs within the polymeric coating of cerium oxide nanoparticles (nanoceria, NC) enhance the colorimetric signal several folds compared to single gold nanoparticles. Furthermore, synergistic interactions between the cerium oxide nanoparticles and several encapsulated GNPs are expected to enhance the plasmonic response of PNC and provide better quantitative detection.

SPR peak maxima shifts of PNC arise in the presence of target analytes due to the enhanced aggregated states of the embedded GNPs, which can be quantitatively measured by UV–vis measurements. PNC was conjugated with target EGP-specific antibody (anti EBOV-mAb) that allows specific binding between the EGP and the PNC, resulting in a detectable SPR change along with a visual readout.

### PNC Synthesis and Characterization

**Figure 1:**
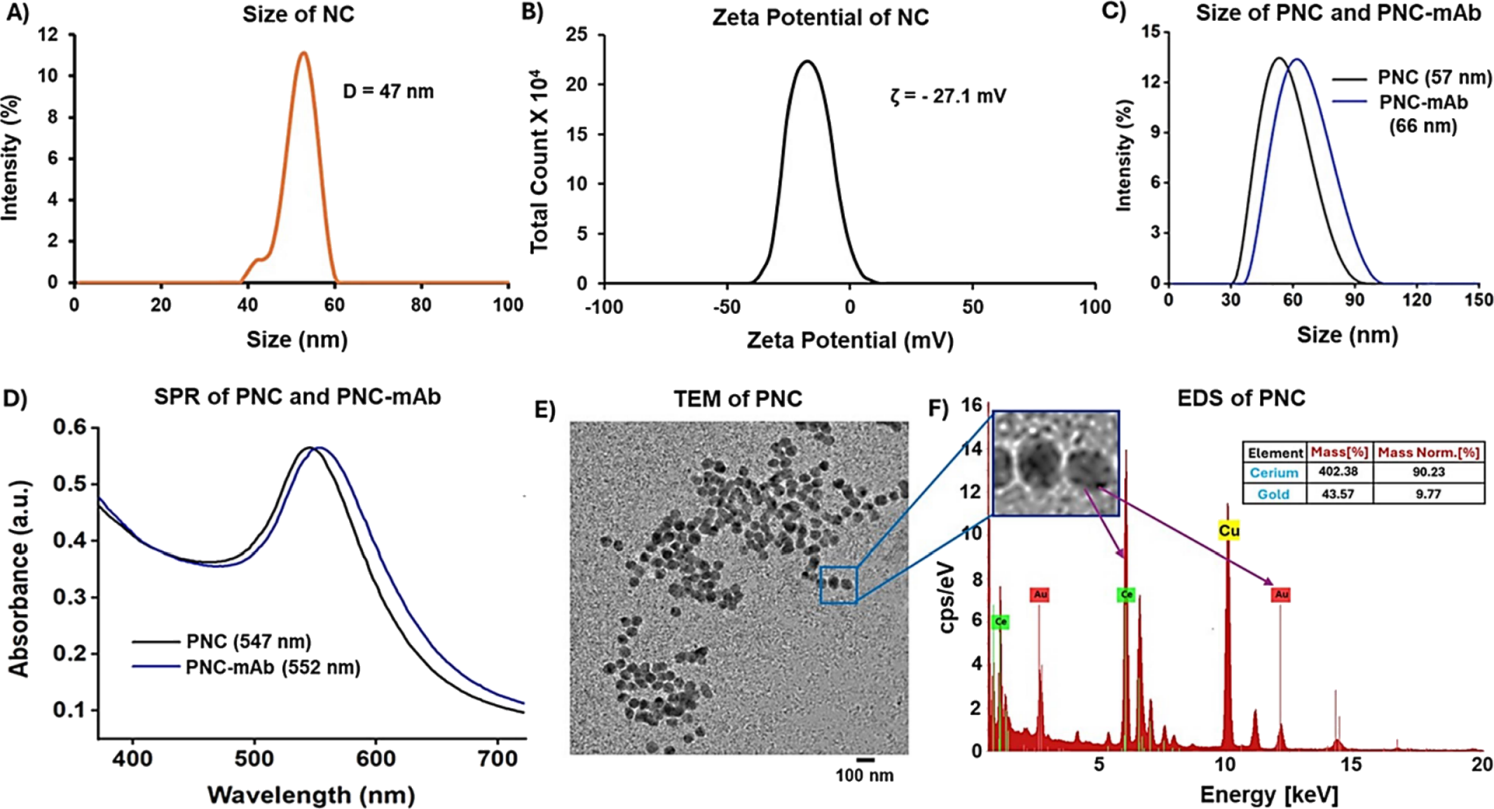
Characterizations of NC and PNC: (A,B) size and zeta potential of the NC. (C,D) Comparison of size and SPR of PNC before and after conjugation. (E) TEM image of PNC (scale bar: 100 nm). (F) Energy-dispersive X-ray spectroscopy (EDS) spectrum of PNC showing elemental compositions of Ce and Au.

For PNC synthesis, poly(acrylic acid)-coated cerium oxide nanoparticles (nanoceria: NC) were first prepared using our previously reported method and characterized using DLS measurements.^36,37^ The hydrodynamic diameter and surface charge of NC were found to be 47 ± 2 nm and −27 ± 3 mV, respectively (Figure 1A,B). Following the synthesis of NC, in situ formulation and simultaneous GNP encapsulations within the PAA coating of NC were conducted in a one-step process by mixing NC with gold chloride solution and boiling it in the presence of sodium citrate. The successful completion of Au^3+^ reduction to Au^0+^ was marked by the appearance of a wine-red color and further used for the assessment of an end point for PNC synthesis. The synthesized PNC was characterized by using several analytical instruments including dynamic light scattering (DLS), transmission electron microscopy (TEM), X-ray photoelectron spectroscopy (XPS), UV–vis, and energy-dispersive X-ray spectroscopy (EDS). The average size of PNC measured by DLS was found to be 57 ± 3 nm (Figure 1C). Furthermore, the size of the nanoparticles was measured over a period of six months at regular intervals, and like our other nanocomposites,^42^ minimal changes were observed.

**Figure 2:**
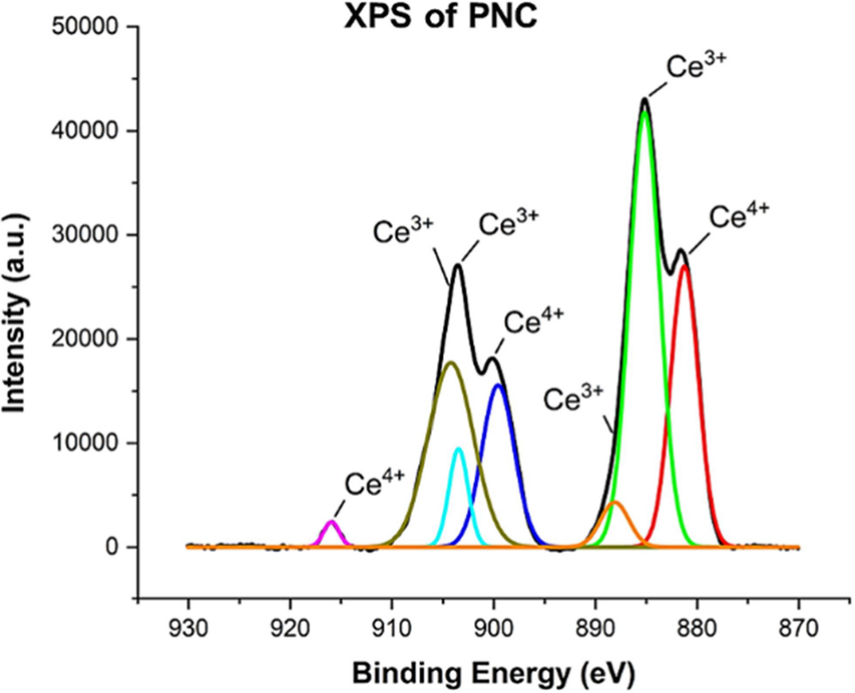
XPS spectra of PNC suggest the presence of mixed valence states of cerium oxide (Ce^3+^/Ce^4+^).

UV–vis experiments on PNC showed λ_max_ at 547 nm (SPR, Figure 1D) that was attributed to the plasmonic absorption of encapsulating GNPs. Compared to GNP’s SPR (λ_max_ = 520 nm) as previously reported,^42^ the absorption maximum of PNC was red-shifted by 27 nm, which further confirmed for their successful encapsulation within the PAA coating of NC. TEM images corresponding to the synthesized PNC indicated the formation of stable, monodispersed, Ce–Au nanocomposites (Inset Figure 1E, scale bar: 100 nm). In addition, EDS studies further confirmed the presence of the elements Ce and Au in the PNC sample. A high-resolution XPS scan of PNC is shown in Figure 2. Following successful PNC synthesis, anti-Zaire IgG (specific antibodies against the Zaire EGP) were passively conjugated onto the surface of PNC. Surface conjugation of antibodies on PNC resulted in an increase in the hydrodynamic diameter from 57 ± 3 nm to 66 ± 2 nm. Corresponding SPR peaks revealed significant shifts, confirming successful conjugation (Figure 1D). In addition, zeta potential values were compared before and after antibody conjugation (Figure S1) for further confirmation.

### Proof-of-Concept Studies Using Functional PNC for Ebola GP Detection

**Figure 3:**
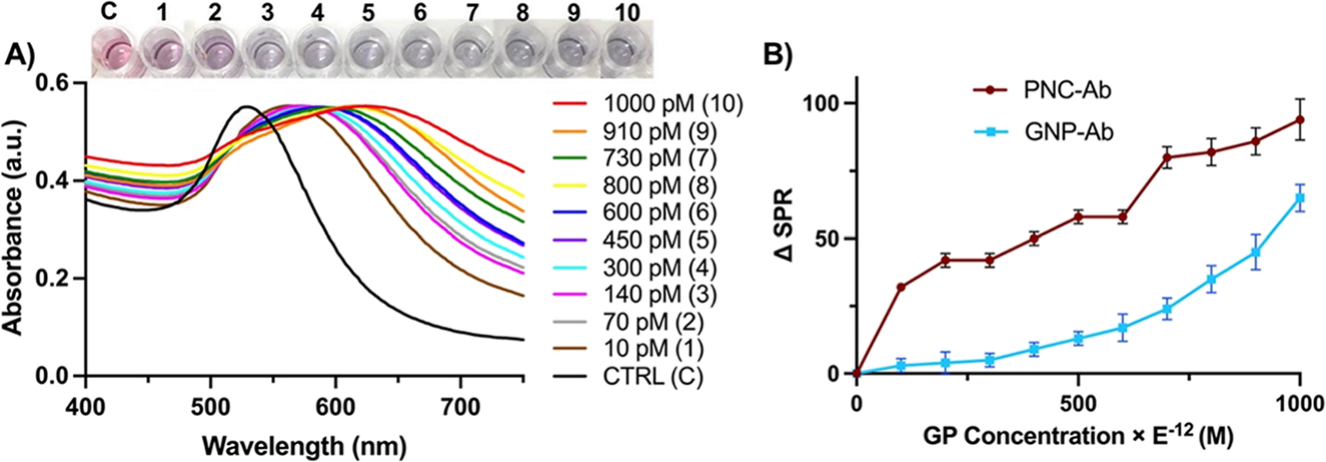
Dual-modal detection of the EGP. (A) Increasing concentrations of EGP were added to PNC-mAb, and the corresponding UV–vis spectra are shown; inset: observed color changes when different amounts of EGP were mixed with the PNC-mAb conjugate. (B) Comparison of changes in absorption maxima (ΔSPR) between PNC-mAb and GNP-mAb in response to EGP addition.

For conducting a detection assay using functional PNC, we selected EGP as the target biomarker. EGP is an important virulence factor for EVD and has been shown to play a vital role for the viral entry into the host cell along with fusion. Hence, it is important to detect this protein in a rapid and specific fashion.^31^ Our initial experiments focused on optimizing the detection conditions and evaluating the dynamic range of the assay. We incubated anti-Zaire IgG-PNC (PNC-mAb) at a constant concentration of 2.0 mM with increasing amounts of EGP (10–1000 pM) spiked into a 1X PBS solution (pH 7.4) for 30 min at 25 °C in a 96-well plate. In the presence of increasing EGP, changes in SPR (Figure 3A) and a visual color change from light blue to dark purple occurred within a few minutes after the addition of glycoprotein (inset, Figure 3A). In contrast, no visual color change was observed in the control sample with no EGP. Additionally, quantitative evaluation of EGP was accomplished by monitoring the corresponding wavelength peak shifts, denoted as ΔSPR (Figure 3B), at each concentration by UV–vis measurements. The results of the SPR plot of PNC-mAb further depict that the λ_max_ shift showed a dose-dependent response at the concentration range of 10–1000 pM of EGP. The calibration curve generated from the SPR responses versus increasing concentrations of EGP indicated a linear response for detection (Figure S2). Using the 3σ/slope method, the limit of detection (LoD) was determined based on the standard deviation of the detection response and the slope obtained from the calibration curve. The LoD obtained from the PNC detection assay was 0.6 pM. By comparing the SPR plot of PNC with that of GNPs under identical conditions (see Figure 3B), it was observed that PNC displayed better plasmonic enhancement; hence, a detection limit of 10 pM (0.7 ng/mL) was seen as compared to 400 pM (28 ng/mL) for GNPs. In the presence of increasing concentrations of EGP, the colloidal status of functional PNC changed from a dispersed to an aggregated state, resulting in subsequent visible color changes due to shifts in the characteristic plasmonic band (SPR) of encapsulating GNPs. These proof-of-concept studies suggest that with PNC, lower limits of EGP detection (LoD) were achieved, which is attributed to the synergistic interactions occurring at the interface between cerium oxide nanoparticles and gold nanoparticles. The performance of the PNC nanosensor for the detection of Ebola antigen was compared with other techniques reported in the literature (Table S1). Moreover, it also demonstrates the simplicity of the colorimetric assay procedure, which does not require extensive sample preparation.

### Specificity of PNC-Based EGP Detection Assay and One-Step Differentiation of Different Subtypes of EGP

**Figure 4:**
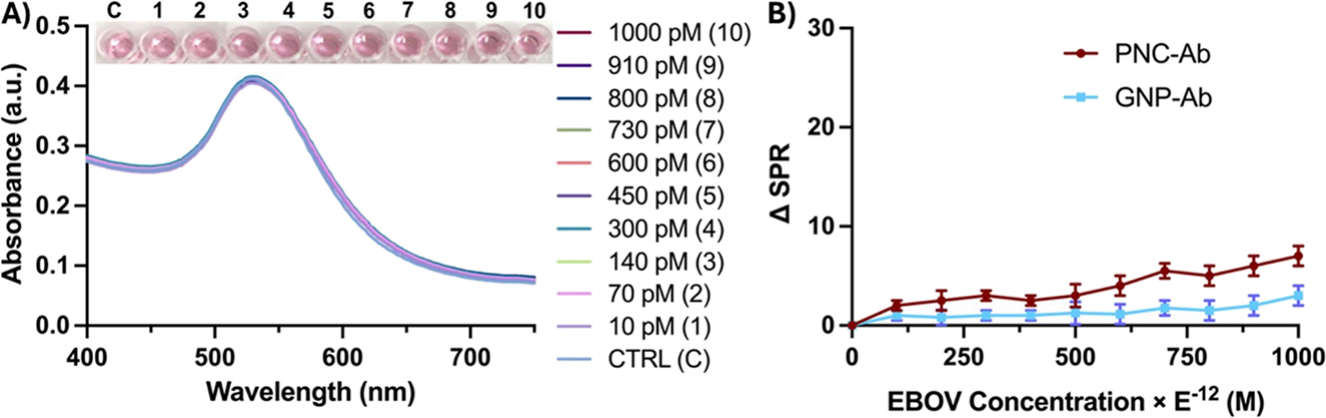
Specificity test: excess free anti-Zaire IgG antibodies were preincubated with increasing concentrations of EGP and then incubated with PNC-mAb. (A) UV–vis absorption maxima and visual-readouts showed minimum interactions. (B) PNC-mAb and GNP-mAb showed a minimum SPR shift.

**Figure 5:**
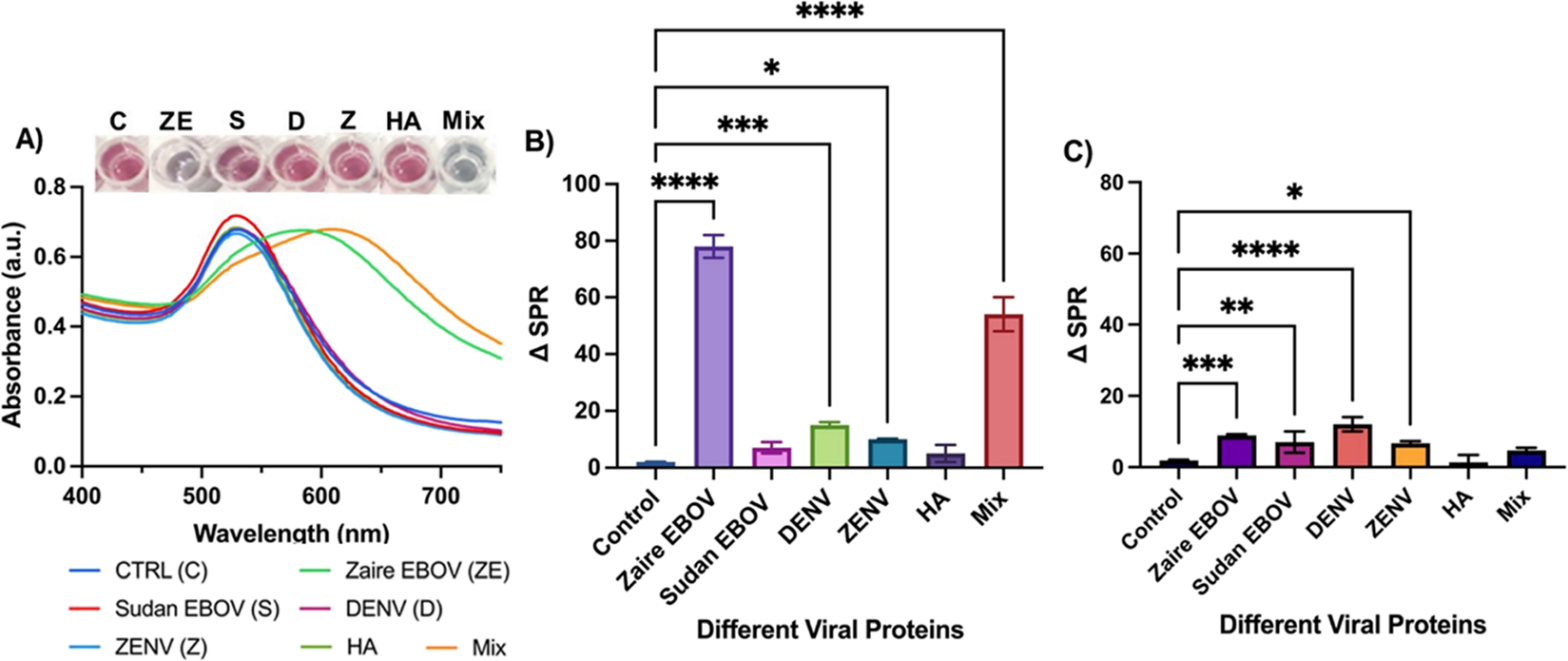
Detection specificity of PNC-mAb in the presence of other nontarget viral proteins. (A) UV–vis data and colorimetric responses, (B) SPR shifts, and (C) SPR responses were obtained when viral proteins were saturated with free anti-Zaire IgG.

To demonstrate the specificity in the interactions between EGP and PNC-mAb, free anti-Zaire IgG (6.0 μM) was preincubated with increasing concentrations of Zaire EGP, followed by the addition of PNC-mAb (2.0 mM). As shown in Figure 4A,B, the addition of free antibodies resulted in minimal changes in ΔSPR values. Likewise, the qualitative readout with no visual color change (Figure 4A) also verified the specificity of the detection assay. Next, we further validated the specificity of the PNC-based detection assay by performing a few control experiments. In our experimental design, other viral proteins including hemagglutinin (HA), full-length dengue (DENV), and Zika (ZENV) virus envelope proteins were included. Sensing performance was evaluated in each case by monitoring ΔSPR and color change after the addition of PNC-mAb. As shown in Figure 5A,B, this resulted in little to no interaction, further validating the selectivity of functional PNC. However, with an experimental solution featuring a mixture (Mix) of EGP and contaminant viral proteins, a significant SPR peak shift and a detectable color change were observed. In addition, antibody saturation studies were also performed by incubating the samples in the presence of free anti-Zaire IgG. Minimal variation in SPR and no color changes were observed under each experimental condition (Figure 5C). Overall, these results indicate that the PNC-based detection strategy is highly selective and sensitive. Furthermore, the PNC-based assay detected targeted EGP even in the presence of other contaminant viral proteins, demonstrating detection sensitivity.

Next, we investigated whether our detection nanosensor platform can distinguish between two or more subtypes of the Ebola virus. The sensitivity and specificity of our detection assay were tested by incubating anti-Zaire IgG-PNC (PNC-mAb, 2.0 mM) with two experimental solutions: 400 pM of Sudan-EBOV and 400 pM Zaire–EBOV. After 15 min of incubation, the quantitative and colorimetric readouts were compared in each case. As shown in Figure 5A,B, only the experimental solution containing Zaire EGP showed detectable binding with anti-Zaire IgG-PNC (PNC-mAb), as manifested by a significant change in the peak wavelength maximum shift of 80 nm along with a visual color transition from wine-red to purple. On the other hand, in the presence of Sudan-GP (Figure 5A–C), no noticeable change in color as well as minimum variation in ΔSPR was recorded. These observations further demonstrate that in addition to the rapid and ultrasensitive detection of EGP, our assay design, employing PNC conjugated with mAbs (specific monoclonal antibodies against Zaire Ebola glycoprotein), could detect and distinguish Ebola-Zaire and Ebola-Sudan in one step in a sensitive fashion. This result is significant as Zaire ebolavirus infections are associated with higher fatality than other subtypes of Ebolavirus.^43^

### Evaluation of the Performance of Functional PNC in Complex Samples

**Figure 6:**
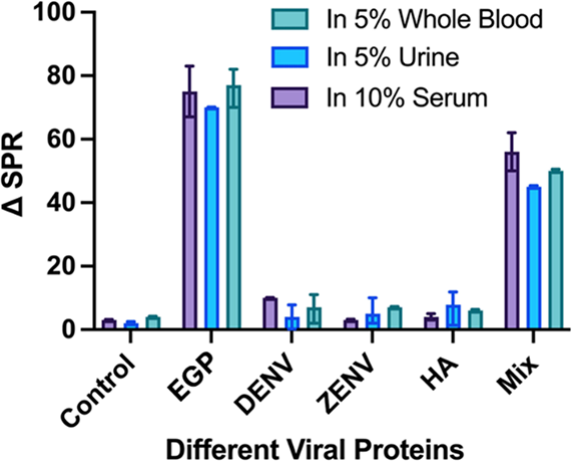
Detection sensitivity in complex media. Specific detection of different viral proteins in 10% serum, 5% urine, and 5% whole blood was performed by comparing SPR shifts.

Determination of assay performance in biological samples is considered critical for analytical sensitivity. The performance of diagnostic assays is often compromised in biological samples due to the presence of several proteins, including casein, albumin, immunoglobulin, and other endogenous salts that result in a high background-to-signal ratio. We evaluated the possible robustness of our detection assay by including 10% serum, 5% urine, and 5% whole blood as complex biological matrices. Initial control experiments were performed using complex biological samples that contained no spiked glycoprotein, and the SPR readings in 1X PBS and biological samples were comparable without any significant difference. The final evaluation of biosensing performance was conducted by spiking 120 pM EGP into 10% serum, 5% urine, and 5% whole blood. These dilutions were implemented to minimize matrix interference during detection. In addition, experiments with other disease biomarkers, including DENV, ZENV, and HA (120 pM), were performed in these complex biological samples. These experiments gave similar results (Figure 6) as in 1X PBS (Control), whereas detection specificity was significant for EGP using the PNC sensing platform. Overall, these experiments demonstrated that the detection of EGP and other biomarkers can be accomplished using our PNC-based detection platform in clinically relevant samples.

### Receptor Screening: Specific Host–Pathogen Interaction

**Figure 7:**
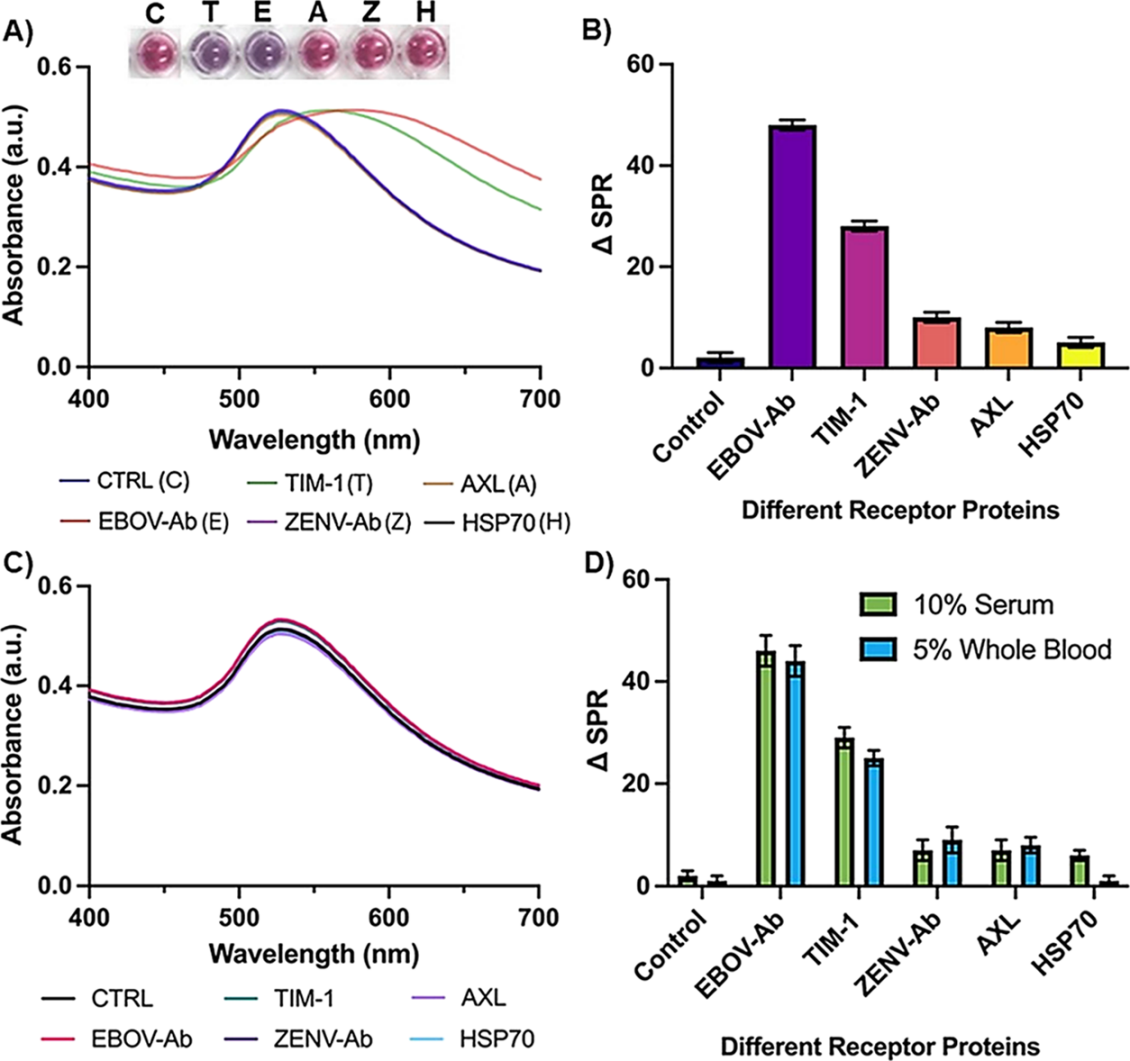
Receptor screening for EBOV was performed by incubating PNC-EGP with various receptor proteins and anti-Zaire IgG (EBOV-Ab). Experiments were assessed using (A) UV–vis data, colorimetric readouts, and a (B) total SPR shift. (C) Competition assay: UV–vis data collected after excess of EGP were preincubated with candidate receptor proteins and antibodies. (D) Detection of different receptor proteins in 10% serum and 5% whole blood using the total SPR shift.

Inspired by our previous findings on the application of magnetic resonance (MR) technology for determining Zika’s candidate receptors,^44^ we wanted to evaluate whether PNC-based nanosensors could also be used as a reliable analytical tool for the effective screening of host receptors for the evaluation of Ebola tropism. The Zaire Ebola virus is known to engage phosphatidyl serine (PS)-dependent and other cellular receptors during its entry into host cells. Previous studies have shown the possible role of TIM-1 and AXL for facilitating Zaire ebolavirus entry and infection.^45,46^ We used EGP-functionalized PNC (PNC-EGP, 2.0 mM) and screened for TIM-1, AXL, and HSP70 (50 μM) as possible candidate receptors. Based on our hypothesis, binding interactions between the glycoprotein and the target receptors should result in a change in ΔSPR that can be quantitatively determined. Additionally, the qualitative and quantitative readouts (SPR and colorimetric) of the interactions of Ebola-Ab and Zika-Ab with EGP-functionalized PNC (positive and negative controls, respectively) provided a baseline comparison that can be used to analyze minute interactions between EGP and proposed receptors (Figure 7A,B). Among all the test solutions, a maximum ΔSPR of 50 nm was seen for the positive control. Interestingly, TIM-1 exhibited maximum binding with EGP among all the selected receptors, as apparent by a significant change in ΔSPR of 35 nm. Additionally, a visual color transition from wine-red to purple was also noticeable in the wells for the positive control (EBOV-Ab) and TIM-1. On the other hand, no significant binding was observed with AXL and Hsp70, with the quantitative and colorimetric readouts similar to that of the negative control (ZENV-Ab), with no detectable change. As an additional control, we also included a control solution with no spiked protein that showed little to no interaction with functional PNC. To explore these interactions further, competition assays were conducted by incubating free EGP with all of the tested solutions for 15 min in 1X PBS (pH 7.4). Next, EGP-functionalized PNC was added, and the wavelength peak shift (ΔSPR) was determined by UV–vis studies. As shown in Figure 7C, these resulted in no SPR peak shifts, which further verifies that the interactions between PNC-EGP and anti-IgG EGP and TIM-1 are specific. Furthermore, similar assays were also performed in complex biological matrices, including 10% serum and 5% whole blood. Comparable results were obtained in each case, as represented in Figure 7D. Taken together, our simple, easy-to-use functional PNC facilitates the screening of receptor candidates in a sensitive, specific, and timely manner.

## Conclusions

In summary, we have successfully demonstrated the development of a new plasmonic nanosensor for the ultrasensitive detection of the Ebola glycoprotein. The successful integration of enhanced plasmonic properties in PNC makes it a significantly more sensitive and reliable tool for early Ebola diagnosis and, at the same time, equally promising for point-of-care tests in resource-limited settings. The experimental outcomes demonstrated the rapid and sensitive detection of EGP in a simple buffer by a dual readout, including ΔSPR and colorimetric detection. The functional PNC could detect EGP at a concentration as low as 10 pM within a few minutes and displayed a broad dynamic range of detection. Our PNC biosensing platform holds a promising alternative considering the lengthy turnaround time of available Ebola detection methods. In addition, using functionalized PNC, a one-step rapid detection and differentiation of Ebola subtypes were achieved. Moreover, the biosensing performance of our easy-to-use functional PNC was not compromised when tested in the presence of complex biological matrices, and the results were comparable to those obtained from a simple buffer. The utility of PNC is not only limited to the early and rapid detection of disease biomarkers but can also be applied to the screening of other potential host receptors of the Ebola virus like AXL, Hsp70, and TIM-1. Overall, the developed new functional PNC offers novel features to be used for field diagnosis and could be applied to the detection of other pathogens.

## Supporting Information Available

Linear detection response of functional PNC for EGP, comparison of the limit of detection using PNC and other available methods, and zeta potential results (PDF)
