# Supplementary material for: Plasmonic Nanoceria: A Plasmon-Enhanced Nanohybrid for Rapid and Sensitive Detection of Ebola Glycoprotein
Source: ACS Appl Nano Mater. 2025 Apr 30;8(18):9604–12. doi: 10.1021/acsanm.5c01649 (PMC12070368; doi:10.1021/acsanm.5c01649)
Supplement: Supplementary file 1 — an5c01649_si_001.pdf [file an5c01649_si_001.pdf]

"Supporting Information"

Plasmonic Nanoceria: A Plasmon-Enhanced Nanohybrid for Rapid and Sensitive Detection of Ebola Glycoprotein

Carissa Sutton,[1] Kristos Baffour,[1] Cassidy Soard,[1] Sneha Ramanujam,[2] Rishi Patel,[3] Santimukul Santra,[1] and Tuhina Banerjee[1,]*

[1]Department of Chemistry and Biochemistry, Missouri State University, 901 S. National Avenue, Springfield, MO 65897, United States of America.

[2] Department of Chemistry, College and Arts and Sciences, Pittsburg State University, 1701 S. Broadway Street, Pittsburg, KS 66762, United States of America.

[3]Jordan Valley Innovation Center, Missouri State University, 542 N. Boonville Avenue, Springfield, MO 65806, United States of America.

*Corresponding author: Tuhina Banerjee, Email: tbanerjee@missouristate.edu

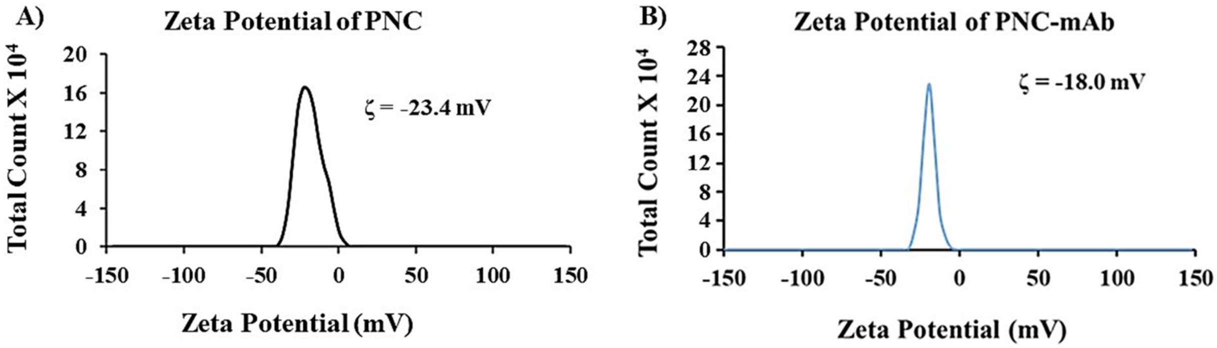

Figure S1. Zeta potentials of **A)** PNC and **B)** antibody functionalized PNC.

Detection Method	Target	Limit of detection	Assay time
Microring resonator sensors[1]	Ebola GP	1.00 ng/mL	40 min
EBOV D4 assay[2]	Ebola GP	100 pg/mL	60 min
single-particle interferometric reflectance imaging sensor[3]	pseudotyped-Ebola virus	5 x10^3 pfu/mL	2 h
Graphene-Based Field-Effect Biotransistor[4]	Ebola GP	0.001 mg/L	Within a few seconds
qRT-PCR assay[5]	EBOV viral RNA	10^3 copies/mL	70 min
upconversion nanoparticles [6]	Ebola virus oligo	7 pM	45 min
ELISA[7]	Ebola GP	0.1 ng/mL	Within 24 hrs
Our method (PNC)	Ebola GP (EGP)	10 pM (0.7 ng/mL)	30 min

Table S1: Comparison between PNC and other available methods for Ebola detection.

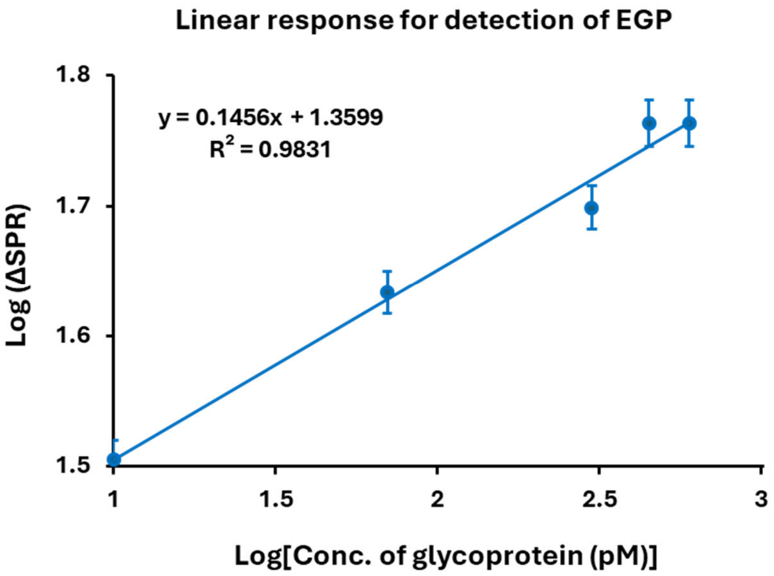

Figure S2. Linear detection response of functionalized PNC towards Zaire-Ebola glycoprotein (EGP) in 1X PBS (pH 7.4).

References:

1) Qavi, A. J.; Meserve, K.; Aman, M. J.; Vu, H.; Zeitlin, L.; Dye, J. M.; Froude, J. W.; Leung, D. W.; Yang, L.; Holtsberg, F. W.; Bailey, R. C.; Amarasinghe, G. K. Rapid Detection of an Ebola Biomarker with Optical Microring Resonators. *Cell Reports Methods* **2022**, *2* (6), 100234.

2) Fontes, C. M.; Lipes, B. D.; Liu, J.; Agans, K. N.; Yan, A.; Shi, P.; Cruz, D. F.; Kelly, G.; Luginbuhl, K. M.; Joh, D. Y.; Foster, S. L.; Heggestad, J.; Hucknall, A.; Mikkelsen, M. H.; Pieper, C. F.; Horstmeyer, R. W.; Geisbert, T. W.; Gunn, M. D.; Chilkoti, A. Ultrasensitive Point-of-Care Immunoassay for Secreted Glycoprotein Detects Ebola Infection Earlier than PCR. *Science Translational Medicine* **2021**, *13* (588).

3) Daaboul, G. G.; Lopez, C. F.; Jyothsna Chinnala; Goldberg, B. B.; Connor, J. M.; M. Selim Ünlü. Digital Sensing and Sizing of Vesicular Stomatitis Virus Pseudotypes in Complex Media: A Model for Ebola and Marburg Detection. *ACS Nano* **2014**, *8* (6), 6047–6055.

4) Arnab Maity; Sui, X.; Jin, B.; Pu, H.; Bottum, K. J.; Huang, X.; Chang, J.; Zhou, G.; Lu, G.; Chen, J. Resonance-Frequency Modulation for Rapid, Point-of-Care Ebola-Glycoprotein Diagnosis with a Graphene-Based Field-Effect Biotransistor. *Analytical Chemistry* **2018**, *90* (24), 14230–14238.

5) Ro, Y.-T.; Ticer, A.; Carrion, R.; Patterson, J. L. Rapid Detection and Quantification of Ebola Zaire Virus by One-Step Real-Time Quantitative Reverse Transcription-Polymerase Chain Reaction. *Microbiology and Immunology* **2017**, *61* (3-4), 130–137.

6) Tsang, M.-K.; Ye, W.; Wang, G.; Li, J.; Yang, M.; Hao, J. Ultrasensitive Detection of Ebola Virus Oligonucleotide Based on Upconversion Nanoprobe/Nanoporous Membrane System. *ACS Nano* **2016**, *10* (1), 598–605.

7) Duan, D.; Fan, K.; Zhang, D.; Tan, S.; Liang, M.; Liu, Y.; Zhang, J.; Zhang, P.; Liu, W.; Qiu, X.; Kobinger, G. P.; Fu Gao, G.; Yan, X. Nanozyme-Strip for Rapid Local Diagnosis of Ebola. *Biosensors and Bioelectronics* **2015**, *74*, 134–141. https://doi.org/10.1016/j.bios.2015.05.025.
